# Osteogenic stimulation of osteoprogenitors by putamen ovi peptides and hyaluronic acid

**DOI:** 10.1186/s13005-023-00380-3

**Published:** 2023-08-08

**Authors:** Jörg Neunzehn, Franziska Alt, Hans-Peter Wiesmann, Benjamin Kruppke

**Affiliations:** 1 Geistlich Biomaterials Vertriebsgesellschaft mbH, Schöckstraße 4, 76534 Baden-Baden, Germany; 2 Technische Universität Dresden, Institute of Materials Science, Max Bergmann Center of Biomaterials, Budapester Straße 27, Dresden, 01069 Germany

**Keywords:** Eggshell peptides, Hyaluronan, Mineralization, Bone regeneration, Osteogenic stimulation

## Abstract

Eggshell peptides (EP) majorly contribute to rapid bone building in chicks, wherefore this paper investigated their potential for stimulating osteogenesis in vitro. In this study, the effects of EP, also called putamen ovi peptides and a combination of hyaluronic acid with EP in cell culture medium were tested towards proliferation, differentiation, gene expression and mineralization of bovine osteoprogenitors and primary human osteoblasts. The influence of EP at concentrations of 0.005 g/L, 0.5 g/L and 0.5 g/L with 0.25% hyaluronic acid was analyzed using immunocytochemical staining of bone-specific matrix proteins, namely collagen type I, osteonectin, osteopontin and osteocalcin, to prove osteoblastic differentiation. Additionally, Richardson-staining was performed. All tests revealed a superior osteoblastic differentiation with EP at 0.5 g/L after 5 days of cultivation. Hyaluronic acid alone showed controversial results and partially constrained osteoblastic differentiation in combination with EP to a level as low as for pure EP at 0.005 g/L. Of particular interest is the osteoblast-typical mineralization, as an important indicator of bone formation, which was measured indirectly via the calcium concentration after cultivation over 4 weeks. The mineralization showed an increase by a factor of 286 during the cultivation of primary human osteoblasts with hyaluronic acid and EP. Meanwhile, cell cultures treated with EP (0.5 g/L) only showed an 80-fold increase in calcium concentration.

The influence of EP (0.5 g/L) on primary human osteoblasts was investigated by gene expression after 2 weeks of cultivation. Microarray and qRT-PCR analysis showed a strongly increased expression of main important genes in bone formation, bone regeneration and the physiological bone remodelling processes. Namely, BMP 2, osteopontin and the matrix metalloproteinases 1 and 9, were present during in vitro osteoprogenitor culture with EP. By explicitly underlining the potential of eggshell peptides for stimulating osteogenesis, as well as emphasizing complex and controversial interaction with hyaluronan, this manuscript is relevant for developing new functionalized biomaterials for bone regeneration.

## Background

For treating large bone defects, autologous bone is considered the gold standard. Because of its limited accessibility as well as the accompanying additional tissue defect, a lot of different materials and methods were developed. The claim on an artificial bone graft substitute is suitable biocompatibility, osteoinductivity and osteoconductivity. Besides researching new materials, a bioinspired approach is to stimulate the bone-building process and to support this in a physiological way. We hypothesize that eggshell proteins incorporated into hyaluronic acid (HA), also called hyaluronan, can stimulate the surrounding cells to regenerate defective tissue.

For supporting metabolic processes, many different factors can be used as stimuli, such as proteins, ions, growth factors and many more. It is recommendable to use reagents which are also prominent in the natural bone. Eggshells are a natural composite, consisting of a rich mineral phase of many relevant mineral trace elements for bone metabolic processes, such as calcium carbonate, calcium triphosphate and magnesium carbonate, which all take up 95.1% of an eggshell’s mass. The positive influence of eggshells in combination with HA on osteoblast activity, cell proliferation, differentiation and metabolic activity of the differentiated cells in comparison to calcium carbonate has been successfully shown earlier [28]. This previous work of Neunzehn et al. was investigating eggshell granulates, wherefore the impact of the mineral phase compared to the impact of the eggshell peptides couldn’t be distinguished. This manuscript is closing this knowledge gap by focusing on the capacity of stimulating osteogenic differentiation of eggshell peptides solely.

Besides the high mineral content, eggshells consist of 3.3% proteins and 1.6% water, defining it as a natural composite. The suitability of eggshells for bone graft substitutes has been intensively researched in different in vivo studies [8, 30, 32]. Eggshell matrix proteins are responsible for the outstanding fast mineralization process of the eggshell, while they also trigger and support the bone development of the chick embryo. Considering these properties as well as biocompatibility, the avian eggshells are more than a calcium carbonate reserve. In terms of osteointegration, cell proliferation, cell migration, and other crucial elements of bone regeneration, this encourages the exploration of eggshell proteins as possible bone formation stimuli. The different eggshell matrix proteins can be classified according to their occurrence with lysozyme, ovalbumin, ovotransferrin and clusterin as egg white proteins, besides ovocleidin 116 or -17 and ovocalyxin-32 or -36 and as uterus and eggshell specific proteins. Some of the aforementioned proteins have the capacity to alter the eggshell calcium carbonate morphology and the rate of precipitation [29, 31, 35, 36]. For bone metabolism, osteopontin (OPN or SPP1) deserves the most attention for its significant role in calcification by increasing osteoblast adhesion onto the matrix while binding to hydroxyapatite [10, 35]. While there is no evidence for toxicity or inflammatory effects, preliminary studies have reported on the biocompatibility and capacity of this protein to attach to the recipient bone [9].

Since another requirement for a bone graft substitute is its applicability and manageability, eggshell proteins need to be implemented in a matrix to be applied in the surgical site. HA has long been an integral part of various therapy concepts in many medical disciplines and has established itself there in recent years. Widely distributed throughout the human body, it serves, among other things, as an important component of the extracellular matrix of many tissues (skin, connective tissue, muscles, tendons, cartilage and bones) and plays an important role in the regeneration and repair of the corresponding tissues [4, 14, 18, 28, 41]. This linear polysaccharide is with its specific biochemical and physical properties an intriguing biomaterial. Incorporated in an aqueous solution, allows one gram of HA to retain up to 6 L of water while maintaining the conformational stiffness. With biochemical properties such as the interaction with different proteoglycans, modulation of inflammatory cells, and scavenging of free radicals, HA is one of the most important substances of different tissues and organ systems [21, 22]. By stimulating the migration and proliferation of different tissue cells and collagen production, hyaluronic acid promotes early wound healing and angiogenesis, has immunomodulatory functions, reduces proinflammatory features, is biocompatible and completely absorbable [2, 20, 26, 38, 40]. The improvement of wound healing process and bone remodelling of HA in combination with other autologous bone, bone graft substitutes and other biomaterials have been verified for in vivo tests and clinical application [7, 11, 34, 43]. Based on these data, the aim of this in vitro study is to investigate the effects of eggshell proteins in different concentrations (0.005 g/L or 0.5 g/L) and in combination with HA as a bone graft substitute with osteoinductive properties. For this purpose, proliferation and molecular biological analysis by microarray and qRT-PCR were investigated after 5 days of primary osteoblast cultivation. Furthermore, based on staining of collagen type I, osteonectin, osteopontin and osteocalcin, differentiation was detected after 14 days, while calcium determination was measured after 4 weeks of cultivation.

## Methods

### Bovine osteoprogenitor isolation

The osteoprogenitors were derived from the periosteum of calf metacarpus, obtained from abattoir tissue and 1 to a maximum of 2 h after sacrifice. The cells originated from one animal. In the regular cell harvesting process, there was no sex-specific selection of animals. The periosteum was cut into 3–6 mm^2^ pieces and transferred into culture dishes. The osteogenic layer of the periosteum specimen were placed face downwards. Osteoprogenitors were allowed to migrate from these specimens for 3 weeks. During this time the explant specimen were cultured in High Growth Enhancement Medium (ICN Biomedicals, Germany) supplemented with 10% fetal calf serum, 250 μg/mL amphotericin B, 10,000 IU/mL penicillin, 10,000 μg/mL streptomycin, 200 mM L-glutamine (Biochrom, Germany) and 10 mM β-glycerophosphate, at 37 °C and 5% CO_2_ in humidified air. The cell culture medium was replaced once a week. Cells of the first passage were used for this study. The osteoblastic character of the osteoprogenitors used in this study was positively shown by immunocytochemical staining (osteopontin, osteocalcin) during cultivation. The cells were harvested by incubation with collagenase (CLS Typ II; Biochrom, Germany) and ready-to-use tyrode solution at pH 7.4, collected and pelleted by centrifugation.

### Osteoprogenitor cultivation with EP – proliferation and differentiation analysis

The obtained osteoprogenitors were resuspended and seeded on the bottom of cell culture dishes (21.5 cm^2^) with densities of 10^4^ cells/cm^2^. Cell counting was performed with CASY Cell counter and Analyser Modell T (Schärfe System, Reutlingen) as suggested by the manufacturer. For further cell cultivation experiments, conditions were chosen equal to those used for the periosteum outgrowth culture. The different groups contained eggshell extracted peptides (provided and concentration recommended by Dermaviduals USA LLC, USA) of concentrations of 0.005 g/L (EP I) and 0.5 g/L (EP II), which were added to the cell culture medium over time of cultivation. A further group contained a combination of eggshell peptides with the concentration of EP II (0.5 g/L) supplemented with hyaluronic acid (0.25%, made from Ostenil®, TRB Chemedica, Molecular weight: 1000–2000 kDa) in the cell culture medium, which is indicated as HAEP. As a negative control, reference cultures were cultivated with cell culture medium without eggshell peptides or hyaluronic acid supplementation. Cultures were examined regularly by light microscopy and the influence of different additives concerning proliferation was measured by cell counting after 5 h, 1, 2, 3, 4 and 5 days of cultivation.

Osteoblastic differentiation was analysed by cultivation until day 14. Therefore, cells were seeded at a density of 6∙10^4^ cells/cm^2^ on culture plates with 56.7 cm^2^ under addition of cell culture medium supplemented as described before. Measurement of immunocytochemical expression patterns of the bone-specific matrix proteins collagen type I, osteonectin, osteopontin and osteocalcin was performed to prove osteoblastic differentiation. Additionally, analysis of the Richardson-staining of bovine osteoprogenitors was performed. All staining procedures were performed in the cell culture dishes. For immunocytochemistry, the cell culture medium was decanted. The specimens were washed three times with phosphate buffered saline (PBS), fixed with cold methanol at -20 °C and subsequently incubated at -20 °C. After removing and drying of residual alcohol, a blocking solution (Dako) was applied for 15 min to saturate non-specific binding sites. Specific antibodies, diluted in blocking solution, were used to detect extracellular matrix proteins by immunocytochemical staining. Therefore, anti-collagen type I (Bio-Trend Chemikalien, Germany; 1:100 in blocking solution), the antibodies anti-osteocalcin and anti-osteonectin (Takara, Japan; each 1:50 in blocking solution) and anti-osteopontin (CHEMICON International, USA; 1:50 in blocking solution) were used and analyzed with secondary antibody Dako EnVision™ + (DAKO; 1:100 in blocking solution). The stained cell cultures were additionally analyzed by light microscopy.

Richardson staining was accomplished with a blue dye (Methylen blue Azur II) as previously described [27]. After methanol-fixation of the cells and decanting the methanol, 2–3 drops of the freshly mixed warm solution were applied and cells were incubated for 2 min at 60 °C. Staining solution was removed and cell layer subsequently washed. Analyzation was performed after drying upside-down in a dark dry chamber.

### Primary human osteoblasts – Biomineralisation by calcium determination

The used primary human osteoblasts belong to a primary cell line of Promocell (Promocell, Germany). Cultivation, passage and harvesting were conducted with products and protocols recommended and distributed by Promocell,

To prove the cellular driven mineral formation by primary human osteoblasts after 4 weeks, the calcium content of the cell layers was examined after dissolution with HCl. The test solution Calcium Arsenazo III consists of 90 mmol/L sodium acetate (pH 5.5) and 348 µmol/L arsenazo. For the measurement the microplate spectrophotometer µQuant-System was used. For mineral formation, 6∙10^4^ cells/cm^2^ osteoblasts were cultivated in 6-well plates and the medium was supplemented with EP I, EP II, and HAEP. The blank medium was used as negative control. After incubation for 4 weeks with weekly medium change, the cells are fixed with methanol and then washed several times. The cell monolayers in the wells were covered with 0.1 M HCl and incubated for five minutes at room temperature. The HCl solution was transferred to sterile Eppendorf tubes, vortexed for 15 s and then centrifuged for three minutes. To determine the mineral content, 300 µL each of the Calcium Arsenazo III Reagent was added to 5 µL sample supernatant in the wells of a 96-well plate. The extinction was determined at a wavelength of 650 nm. Extinctions were assigned to the amount of calcium containing mineral formed by the cell.

### Primary human osteoblasts – Molecular biological analysis by microarray and qRT-PCR

For a detailed analysis of the influence of the pure higher concentrated putamen ovi proteins (EPII), a gene expression analyses of primary human osteoblasts (Promocell, Germany) was used. After a short incubation time, the cells were seeded into 75 cm^2^ cell culture bottles with a cell density of 10^4^ cells/cm^2^. After three days, the cells were passaged, and transferred into the wells of 6-well plates with a cell density of 2∙10^4^ cells/cm^2^. In order to evaluate the effect of the isolated eggshell peptides (EP) on the primary human osteoblasts, the peptides were added to the cell culture medium with a concentration of 0.5 g/L and the cells were incubated at 37 °C and 5% CO_2_ for five days. Similar cell cultures from the same batch and passage, provided with untreated culture medium served as the negative control. After reaching confluence (at day 5), the cells were detached from the cell culture dish and centrifuged for three minutes. The supernatant was discarded and the cells were stored in 700 μL of Qiazol (Qiagen, Germany) in cryotubes at -80 °C.

The RNA was isolated from the cells using the miRNeasy kit (Qiagen, Germany) according to the manufacturer's instructions. The quality and quantity of RNA obtained was determined by photometric measurements (NanoPhotometer™, Implen, Germany) and by the use of a bioanalyzer (RNA Nano 6000 Lab-Chip® Kit, Agilent Technologies, USA).

The cDNA was synthesized using a cDNA Synthesis Kit (Roche, Germany), according to the manufacturer´s data. 1 μg of RNA was used as starting material for cDNA synthesis. Subsequently, the NimbleGen One-Color DNA Labeling Kit (Roche, Germany) was used to label the cDNA with Cy3. The concentration of the labeled cDNA was determined using the NanoPhotometer. Subsequently, the hybridization (NimbleGen Hybridization Kit) and the required washing (NimbleGen Wash Buffer Kit) were carried out according to the manufacturer's instructions. The arrays used for the analysis were scanned with the NimbleGen MS 200 Microarray scanner (pre-scan: 40 μm, scan: 2 μm). The results of the measurements were analyzed, normalized and then statistically evaluated.

The results of the cells treated with EP were compared with those of the untreated control cells. The analysis was performed to identify the top 100 up- and down-regulated genes. The result is a matrix holding the gene name, the *p*-value, and the x-fold change. The ranking of this matrix is according to the x-fold change. In this analysis the *p*-value cutoff is at 5%. Furthermore, the heat map shows a two-dimensional clustering of the top one hundred genes, where intensities are represented by the colors red and green, for high and low intensities, respectively.

For further validation of individual genes, a qRT-PCR analysis (quantitative real-time polymerase chain reaction) was performed. Therefore, RNA of the samples was isolated with the miRNeasy Mini Kit (Qiagen, Germany). DNA digestion was performed to eliminate genomic DNA. The samples were treated in accordance with the Machery-Nagel protocol for rDNase digestion in solution with the rDNase set (Machery-Nagel, Germany). The samples were incubated for 10 min at 37 °C and the RNA was precipitated according to the protocol of the manufacturer with ethanol and finally taken up in nuclease-free water. Subsequently, the isolated RNA was transcribed into cDNA using the transcriptor High Fidelity cDNA Synthesis Kit (Roche, Germany). In parallel to the analyzed samples, a negative control without reverse transcriptase is additionally prepared in order to check the success of the reverse transcription. The reaction batches were incubated for 30 min at 55 °C in a thermocycler (GeneAmp PCR System 9700, Applied Biosystems, USA). Subsequently, the transcriptor High Fidelity Reverse Transcriptase was inactivated at 85 °C for a period of 5 min. Until further use the cDNA was stored at -20 °C.

The LightCycler 480 Instrument II, 96-well (Roche, Germany) and the LightCycler® 480 software version 1.5 (Roche, Germany) were used to quantify the expression levels. The samples were applied to 96-well plates (PCR 96-well TW-MT plate, white low profile, semi-skirted, DNase-, RNase-free, for LC 480 I and II (Biozym, Germany)) and quantified by LightCycler 480 SYBR Green I Master (Roche, Germany)) according to the manufacturer's instructions in a reaction volume of 20 μL. The LightCycler 480 Control Kit was used to check the successful quantification. The gene expression was validated using triplicates of all samples, whereby 1:20 dilutions of the samples were quantified. The primers required for the qRT-PCR were designed according to the usual criteria and synthesized by MWG (Eurofins, Germany). In this study, established housekeeping genes of human osteoblasts GAPDH (HKG 1) and HPRT were used.

### Statistical analysis

All samples were treated and measured as triplicates and given as mean ± standard deviation. A one way analysis of variance (ANOVA) with Bonferroni correction was applied for statistical analysis with the aid of SigmaPlot®12 (Systat Software, Germany). *P* values < 0.05 were considered as significant and indicated by one asterisk (*). Furthermore, *P* < 0.01 is indicated by ** and *P* < 0.001 by ***. The validity and applicability of the different groups and the corresponding values for the statistical analysis were automatically demonstrated by Sigma Plot®12.

## Results

In this study the effects of different concentrated EP and a combination of hyaluronic acid and EP were tested towards the proliferation, differentiation, gene expression and mineralization of bovine osteoprogenitors and primary human osteoblasts. All osteoprogenitors used in the cell culture assays of this work were checked daily by light microscopy to preclude culture contamination or unphysiological changes in morphology.

### Osteoprogenitor proliferation under EP administration

**Fig. 1:**
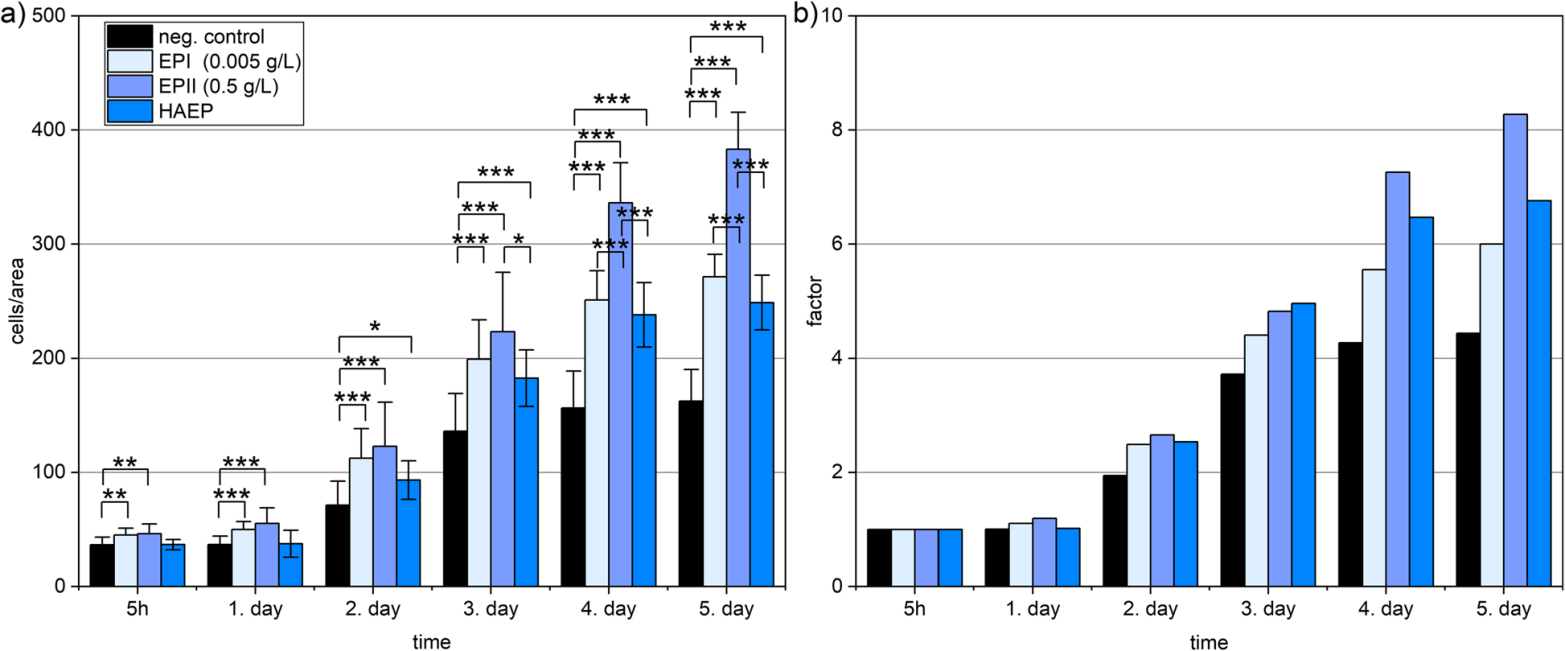
Cell proliferation over five days with an initial cell count of 10^4^ cells/cm^2^ (**a**) with the untreated medium (control), 0.005 g/L eggshell peptides (EP I), 0.5 g/L EP (EP II), and medium supplemented with hyaluronic acid and 0.5 g/L EP (HAEP). Normalized cell counts (**b**) are calculated with the initial cell count after 5 h set as 100%

The proliferation profiles of the osteoblasts treated with the different supplemented media (Control, EP I, EP II and HAEP) are given as absolute numbers over five days ( \* MERGEFORMAT Fig. 1 a) and normalized (to the cell count after 5 h) cell counts ( \* MERGEFORMAT Fig. 1 b). For all groups, the proliferation curves show continuous and significant increase in cell count with all treated groups showing a higher proliferation than the control after two days. During the test period of five days, the cell number of the EP II-treated cell cultures increased to the highest degree by a factor of 8.4, while proliferation factor of the control was only 4.4.

### Immunocytochemical detection and cell differentiation

To investigate the influence of the proteins on cell differentiation of osteoprogenitors histological and immunocytochemical staining of the cultivated cells were performed.

#### Richardson staining

The dye used stains basophilic and osmophilic structures of the cells in blue and metachromatic cell components in red-violet. It is used to assess the phenotype, the density and the distribution of the cells. After an incubation period of two weeks, the cultures showed significant differences in terms of their cell density.

**Fig. 2:**
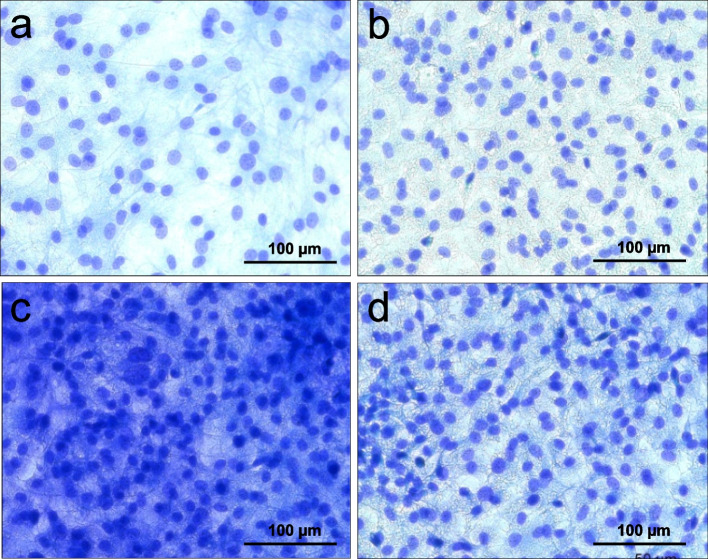
Osteoblast staining according to Richardson. Control (**a**), EP I (**b**), EP II (**c**), HAEP (**d**)

The control group shows compared to the other cultures the lowest cell density and a relatively low level of extracellular matrix (Fig. 2 a). The second lowest number of cells and extracellular matrix is present in the case of low-concentrate peptides EP I (Fig. 2 b). In contrast, cells of sample HAEP (Fig. 2 d) show a highly closed cell monolayer with a significantly prominent intercellular network. By far the highest cell density is present for cell cultures treated with EP II (Fig. 2 c). Here, the cells are allocated in close vicinity with a deep blue-stained network formed between the individual cell nuclei. Regarding the phenotype, the size of the cells and the shape of the cell nuclei do not show significant differences.

#### Expression of collagen type I

**Fig. 3:**
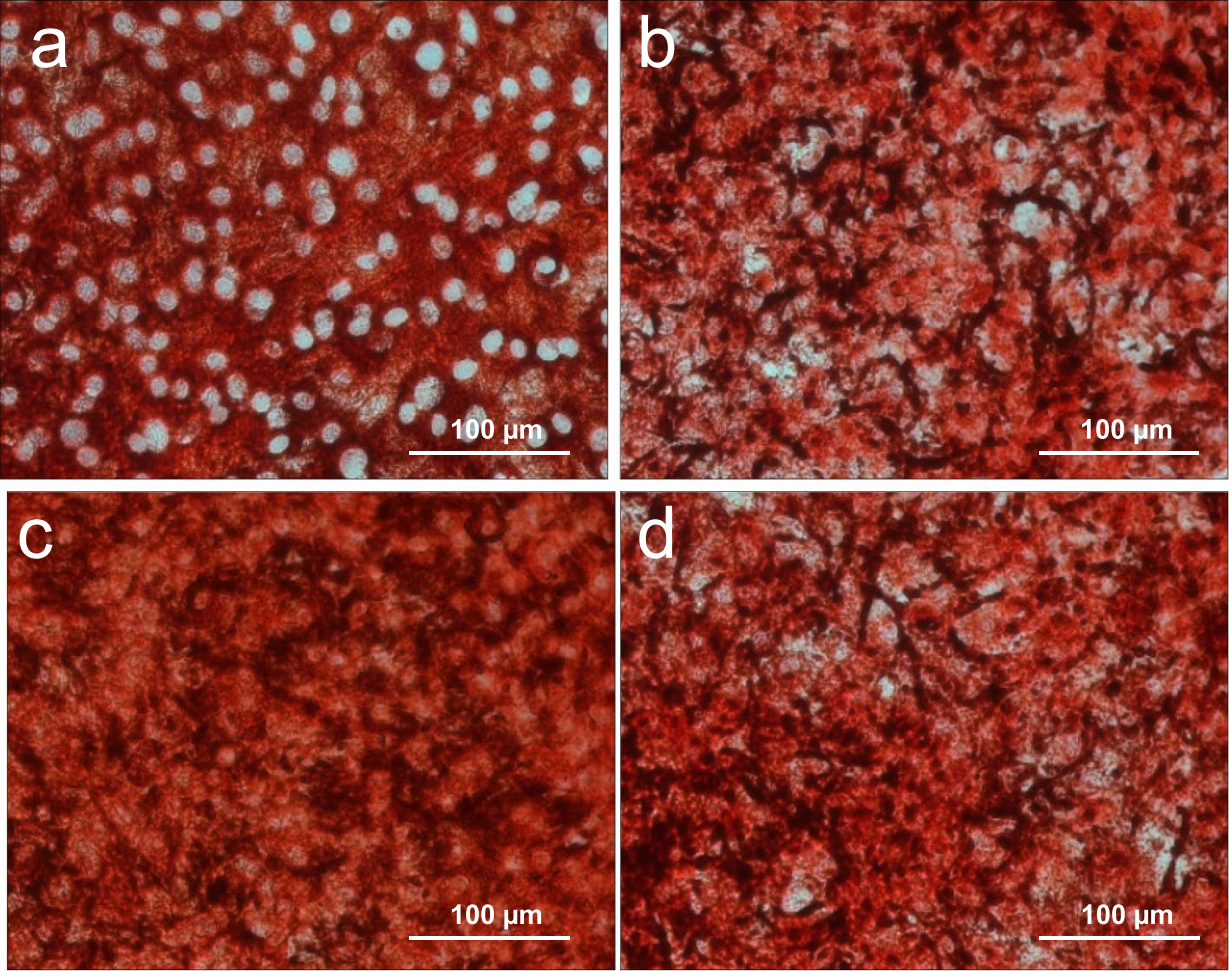
Immunocytochemical staining of collagen type I. Control (**a**), EP I (**b**), EP II (**c**), HAEP (**d**)

Collagen type I is a fibrillar structure protein of the extracellular matrix, which is expressed by osteoblasts. Staining with anti-collagen type I ( \* MERGEFORMAT Fig. 3) shows the differences in collagen expression.

Cells in the control group show a clearly pronounced extracellular collagen network ( \* MERGEFORMAT Fig. 3 a). The cell nuclei can be distinguished with ease from their surroundings. A higher density of the stained structures is visible in cultures of the EP I group and HAEP group ( \* MERGEFORMAT Fig. 3 b and d, respectively). In these groups, the collagen network is expressed in a degree that individual cells cannot be distinguished from each other, as it is the case in the control culture. In contrast, the cells of the group EP II show a very high extracellular assembly of the protein in form of a network of fibrous collagen type I ( \* MERGEFORMAT Fig. 3 c). The density and strength of this network is much more pronounced than in the cell cultures of the other groups. The cells cannot be distinguished from each other.

#### Expression of osteonectin

The protein osteonectin is a non-collagenous glycoprotein, secreted by osteoblasts into the extracellular matrix.

**Fig. 4:**
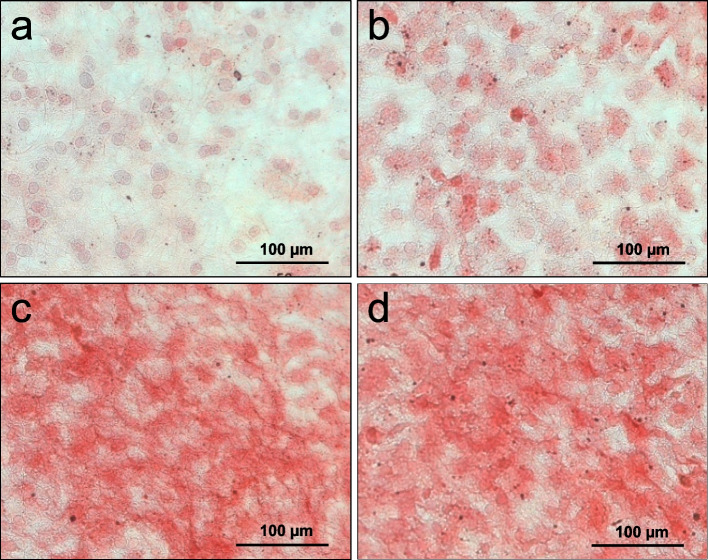
Immunocytochemical staining of osteonectin. Control (**a**), EP I (**b**), EP II (**c**), HAEP (**d**)

As a result of the immunocytochemical staining of osteonectin after two weeks of culture, the protein was detected with almost equal intensity in the control and the EP I culture ( \* MERGEFORMAT Fig. 4 a and b, respectively). The EP II and HAEP groups ( \* MERGEFORMAT Fig. 4 c and d, respectively) show a considerably more intensive staining. Once again, the intensive stain makes it difficult to distinguish individual cells.

#### Expression of osteopontin

**Fig. 5:**
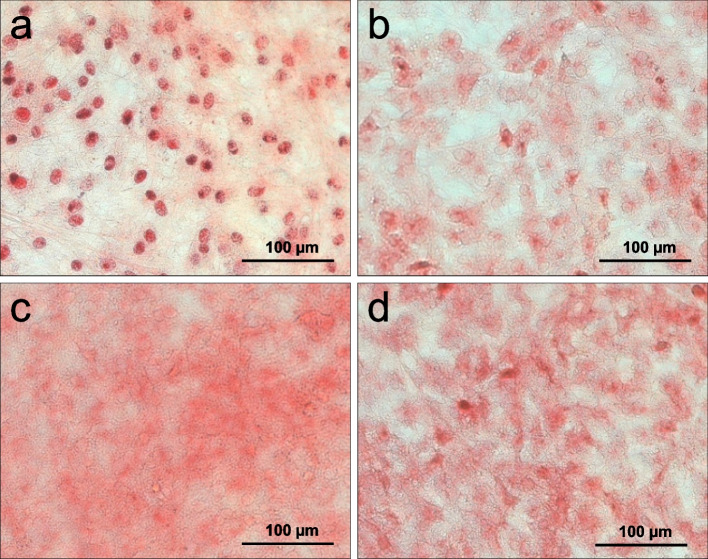
Immunocytochemical staining of osteopontin. Control (**a**), EP I (**b**), EP II (**c**), HAEP (**d**)

Osteopontin is a glycoprotein and is responsible for bone tissue formation and the preservation of the bones. It binds hydroxyapatite and is hence involved in the bone matrix formation. The results of the immunocytochemical study concerning the expression of this protein show clear differences in the various cultures ( \* MERGEFORMAT Fig. 5).

In the control culture, the cell nuclei are clearly stained with poor extracellular osteopontin expressions ( \* MERGEFORMAT Fig. 5 a). The expression in HAEP culture ( \* MERGEFORMAT Fig. 5 d) and EP I culture ( \* MERGEFORMAT Fig. 5 b) are quietly similar. The EP II culture shows most intensive osteopontin staining ( \* MERGEFORMAT Fig. 5 c), indicating the highest protein synthesis. A strong extracellular arrangement of osteopontin is visible.

#### Expression of osteocalcin

**Fig. 6:**
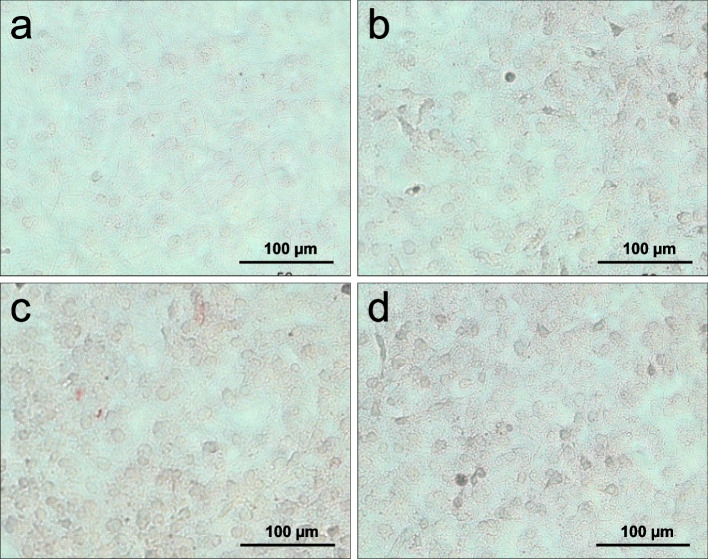
Immunocytochemical staining of osteocalcin. Control (**a**), EP I (**b**), EP II (**c**), HAEP (**d**)

The control culture shows the lowest staining of osteocalcin compared to the other samples ( \* MERGEFORMAT Fig. 6 a). Once again there is a ranked intensity of staining, with group EP I ( \* MERGEFORMAT Fig. 6 b) showing a slightly more pronounced intensity than the control group. Furthermore, the intensity of staining is seen more clearly in the samples of EP II ( \* MERGEFORMAT Fig. 6 c) and HAEP ( \* MERGEFORMAT Fig. 6 d) with weakly stained intracellular structures. In the extracellular matrix of cells only occasionally accumulations of the protein are visible.

#### Biomineral formation

**Fig. 7:**
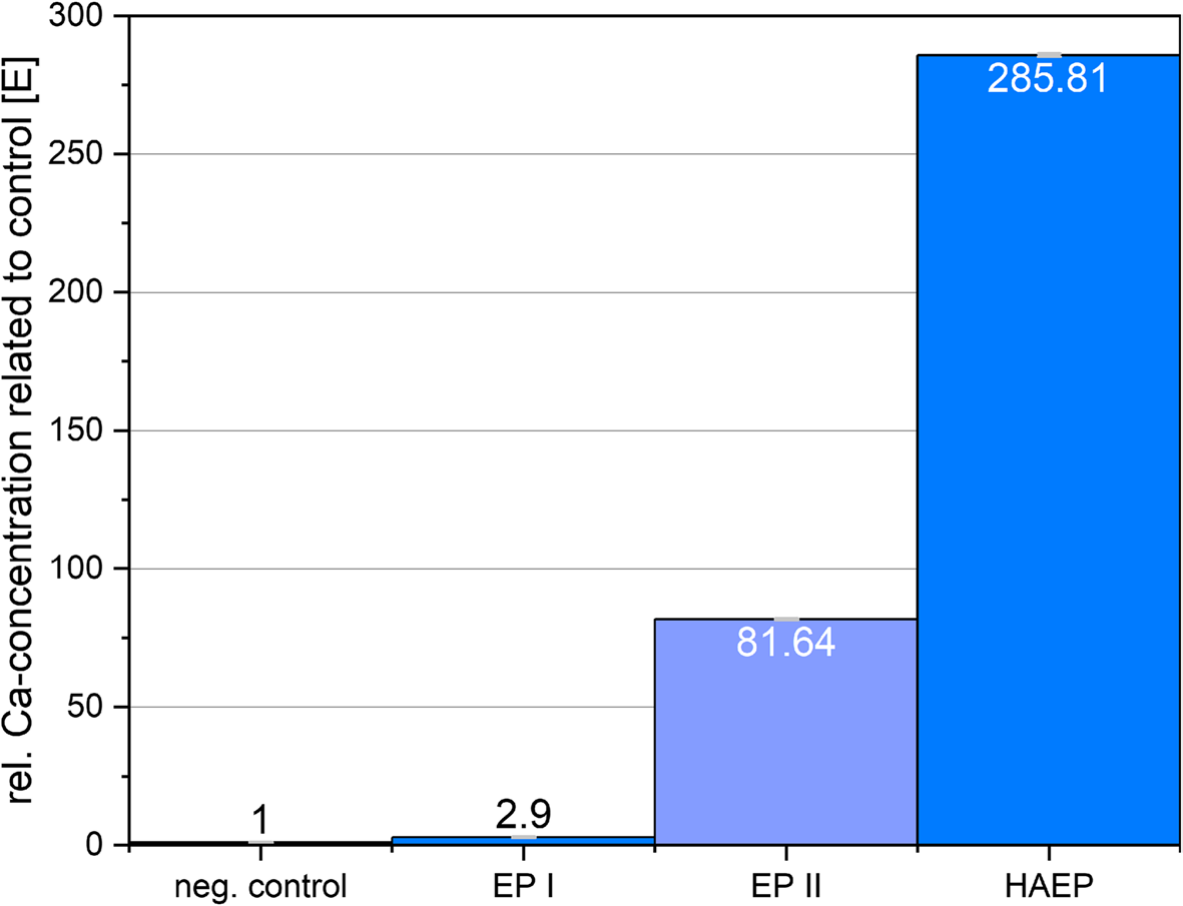
Extinction of stained calcium ions for determination of the cell-bound calcium after 4 weeks at a starting population of 60,000 cells/cm^2^ (normalized to the value "1" of the control culture). Calcium concentration factors of the groups: EP I: 2.9; EP II: 81.64; HAEP: 285.81 are integrated in the bars of the groups

To investigate the peptide-induced biomineralization of primary human osteoblasts a spectrometric calcium analysis was performed. The mineral formation over a period of four weeks for the groups EP I, EP II and HAEP was measured and compared to the negative control without additives ( \* MERGEFORMAT Fig. 7).

Only slight increase of mineral formation over the negative control (factor ≈3) was present at low concentration of EP (EP I). The mineral contend of cells cultured with EP II is still very high compared to the negative control (factor ≈80). The calcium concentrations of the samples EP I and EP II are below the values of the peptides in combination with hyaluronic acid (HAEP) by far. The concentration of organic and inorganic bound calcium in the culture is about 286 times higher than in the control culture without any proteins and shows therefore the strongest effect on mineralization.

### Gene expression of primary human osteoblasts

#### Microarray analyzes

**Fig. 8:**
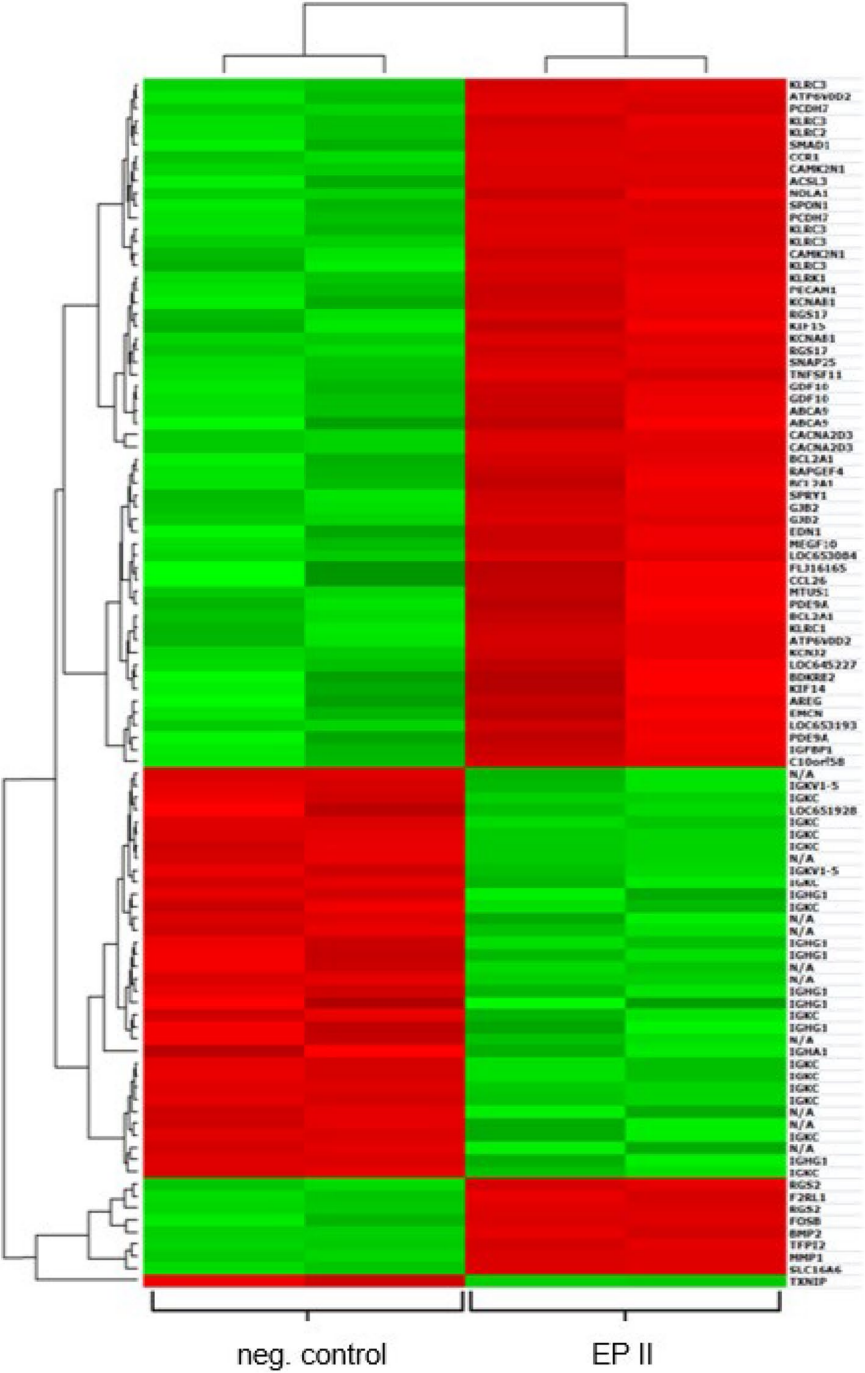
Evaluation of the microarray analysis of the primary human osteoblasts treated with EP II compared to control cultures (untreated). The "heat map" shows the two-dimensional representation of the differently expressed genes. The "red" marked lines represent "down-regulated" gene expression

By means of microarray technology, the impact of the isolated EP on gene expression of primary human osteoblasts was investigated. As a result of the experiments described in the previous section, the peptides were added to the cell culture medium with the most effective concentration of 0.5 g/L and compared to EP free reference. DNA recovered from the cells of both cultures were compared with each other and the 100 genes, which were influenced the most by addition of the peptides, are represented in the heat map in \* MERGEFORMAT Fig. 8. They were split into 65 up-regulated and 35 down-regulated genes.

Visible are four columns either green or red-colored. The two left columns represent the control samples of the “untreated” cells. The two right columns represent the results of the “EPII-treated” group. The results of hierarchical cluster analysis (dendogram on the left side in \* MERGEFORMAT Fig. 8) illustrates the similarities of the genes to each other and is associated with the level of expression of the gene direction. Furthermore, the color-coding indicates, that more genes are up-regulated by primary human osteoblasts treated with EP II compared to the control.

In the heat map, 35 genes are indicated as down-regulated ones in case of EP II treatment (red indicated genes in \* MERGEFORMAT Fig. 8 right side of the “heat map”). Of these, 23 can be directly address to the family of immunoglobulins. The other twelve are not directly identified, but according to the dendogram, they are very similar to the immunoglobulins. The immunoglobulin family is related to the immune response, which would be important for later application, but is not the focus of this manuscript. For this reason, the aforementioned downregulated genes related to immunoglobulins will be neglected. The up-regulated genes presented are primarily related to osteogenic differentiation. Aiming at the impact of EG on osteogenic differentiation, the related genes are discussed in more detail below.

Out of the increased expressed genes the 19 most significant influenced genes are listed in \* MERGEFORMAT Table 1. In addition to the genetic code, the gene names, the *p*-value to each gene and “fold change” is assigned. The latter value represents the factor by which the level of expression of the gene is different from the control.

**Table 1:** Representation of higher-expressed genes with assignment of the expression factor and the corresponding *p*-value

Gen code	Gen name	*p*-Value	Fold change
[CACNA2D3]	calcium channel, voltagedepend, alpha 2/delta 3 subunit	3.44e-04	7.48
[FOSB]	FBJ murine osteosarcoma viral oncogene homolog B	3.07e-02	7.23
[GJB2]	gap junction protein, beta 2, 26 kDa (connexin 26)	2.07e-03	5.94
[EDN1]	endothelin 1	2.50e-02	5.70
[IL8]	interleukin 8	8.18e-03	5.21
[BCL2A1]	BCL2-related protein A1	7.04e-03	4.88
[PCDH7]	BH-protocadherin (brain–heart)	2.07e-03	4.59
[KCNJ2]	potassium inwardly-rectifying channel, subfamily J, member 2	1.30e-02	4.52
[BMP 2]	bone morphogenetic protein 2	9.07e-03	4.44
[TNFSF11]	tumor necrosis factor (ligand) superfamily, member 11	6.50e-03	4.39
[EMCN]	endomucin	1.02e-02	4.31
[AREG]	amphiregulin (schwannomaderived growth factor)	3.37e-02	4.30
[CCR1]	chemokine (C–C motif) receptor 1	1.26e-02	4.05
[MMP 1]	matrix metallopeptidase 1 (interstitial collagenase)	1.24e-03	3.90
[KLRC3]	killer cell lectin-like receptor subfamily C, member 3	4,35e-02	3.78
[GDF10]	growth differentiation factor 10	1.26e-02	3.65
[PECAM1]	platelet/endothelial cell adhesion molecule (CD31 antigen)	3.66e-03	3.51
[SPON1]	spondin 1, extracellular matrix protein	3.05e-02	3.21
[MEGF10]	multiple EGF-like-domains 10	3.62e-03	3.13

### Validation of the microarray analysis using qRT-PCR

**Fig. 9:**
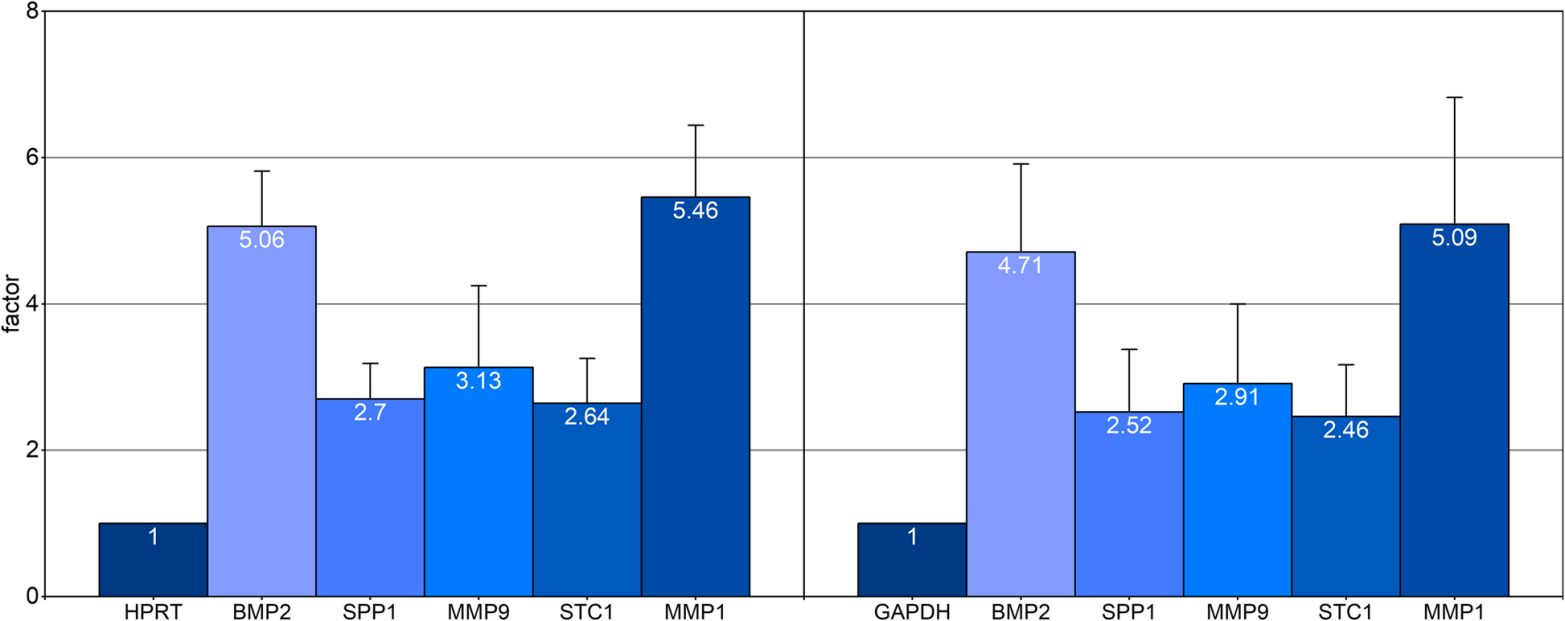
Expression of the genes BMP 2, SPP 1, MMP 9, STC 1 and MMP 1 of the “EPII-treated” group determined by qRT-PCR as measured by the housekeeping genes HPRT (left) and GADPH (right) (standardized to the value “1”) on primary human osteoblasts. The expression factors of the different genes are shown in the respective bars

To confirm the results of the microarray analysis, qRT-PCR was carried out for selected genes on “EPII-treated” group. Of particular interest are genes that have a direct influence on the osteogenic differentiation of primary human osteoblasts or the bone remodelling process. Therefore, BMP 2 and MMP 1 (listed in \* MERGEFORMAT Table 1) MMP 9, stanniocalcin 1 (STC 1) and osteopontin (OPN or SSP 1) were investigated. Expression of these genes in the treated primary human osteoblast culture was compared to both housekeeping genes HPRT and GAPDH (standardized to “1”) individually ( \* MERGEFORMAT Fig. 9).

In both cases the genes BMP 2 and MMP 1 showed an increased value, confirming the microarray analysis. Likewise, the increased expression of SPP 1, MMP 9, and STC 1 are demonstrated by the qRT-PCR analysis as clear trend, but without significant differences.

## Discussion

A prospective use of EP in artificial bone replacement materials or scaffolds with the aim of stimulating bone healing requires a thorough characterization of the effects of the proteins at the cellular level. In this study, in vitro testing of differently concentrated peptides revealed a significant effect on bovine osteoprogenitors and primary human osteoblasts and was investigated by proliferation, osteogenic differentiation expressions in means of Richardson staining, collagen type I, osteonectin, osteopontin, osteocalcin, biomineral formation and microarray analyzes, supported by qPCR.

The proliferation could be increased significantly for all peptide concentrations on bovine osteoprogenitors. No appreciable increase in proliferation from EP I compared to HAEP with day 4 was shown, which indicates the maximum capacity for increasing the cell proliferation of peptide concentration of 0.005 g/L. The contra-intuitive stagnation of proliferation after interaction with HAEP, underlines the importance of investigating the impact of the carrier material on the implemented active ingredients. It can be assumed, that the incorporated EP couldn’t be released as fast as for pure application.

From the literature, the average turnover of osteopontin in healthy humans was found to be 11 min [12], but no evidence of the biological half-life of the other peptides in vitro, nor their direct effect on osteoprogenitors are known. The use of HA in combination with the peptides (HAEP) caused a significant increase in proliferation compared to the negative control, but the cell count was significantly lower than in the EP II group without HA. The most promising concentration of HA for in vitro use is still unknown and in focus of investigations. Li et al. performed an in vitro test in rat calvaria defects with different HA concentrations (1 g/L, 25 g/L, 50 g/L and 75 g/L). The highest bone volume/tissue volume ratio, determined by µCT, as well as the largest area of woven bone with the best vascularization, determined by histological analyses, was observed at 75 g/L [23]. Huang et al. obtained best results at an active compound concentration of 1 g/L HA, which is 2.5 times lower than the amount added in the present study, while investigating 0.5, 1.0, and 2.0 g/L HA. This indicates, that there are more factors on the efficiency of HA, than the concentration. Studies of various authors showed controversial effects of the molecular weight of HA for proliferation and differentiation [5, 16, 33, 39, 44]. In their direct comparison of the molecular weights of HA (60 kDa, 900 kDa, 2300 kDa) no differences regarding cell growth were detected, wherefore the selected HA should have shown equivalent results. In comparison, Zhao et al. reported a promoting influence of low molecular weight (< 10^3^ kDa) on proliferation of rabbit bone-marrow-derived stem cells, while a high molecular weight (> 10^3^ kDa) supported expressions of RUNX-2 and osteocalcin. The selected HA (Ostenil®) at 1000–2000 kDa showed equivalent results to Zhao et al. in terms of proliferation.

The expression patterns of osteonectin and osteocalcin in HAEP-treated cultures were at the same level as in EP II and increased compared to the untreated control. The osteogenic differentiation of the cells under the addition of peptides was most prominent in cultures treated with high concentrated peptides (EP II). This was visualized with staining of the extracellular matrix (according to Richardson), collagen type I and osteopontin. The synthesis and expression of these structural proteins also indicate an enhanced formation of the organic extracellular matrix. Furthermore, the expression of EP I treated cultures showed a slightly increased expression compared to the negative control only. Thus, the low peptide concentration in EP I again leads to the conclusion that there was no sufficient stimulation by the proteins over the entire cultivation period. Overall, HAEP has to be associated with a rather low to moderate stimulation of osteogenic markers, which are somewhat comparable to the values of EP I.

In this regard, and with regard to the results of the proliferation test, the result of the biomineral formation is of particular importance. A particularly strong influence of the peptides on biomineral formation was present. As an endpoint determination of osteoblast differentiation, the experiment showed different mineralization effects depending on the applied peptide concentrations. After four weeks of drug application, the amount of bound biomineral in the cells for lower concentrated EP I (0.005 g/L) was only slightly above the control values. The values of the calcium content of the cultures with higher concentrated EP II (0.5 g/L) showed a significant increase in the mineral content by a factor of 81 compared to the untreated culture medium of the control. The combination of HA and the highly concentrated peptides resulted in a manifold increase in intercellular calcium concentration compared with the cell cultures treated with the peptides solely. Compared with the control culture the value increased by a factor of approximately 286. The results in terms of supplying the cells with HA, and the resulting stimulation of osteogenic cell differentiation are consistent with the aforementioned findings of Huang et al. The positive influence of HA on mineralization with varying degrees of intensity was observed by several groups investigating HA as sole material or on substrates [1, 16, 24].

The molecular genetic analysis and its evaluation by the use of microarrays and qRT-PCR on primary human osteoblasts underlines the stimulating influence of EP on osteogenic differentiation, as well as the general enhancement of tissue differentiation. This is shown by a variety of significantly up-regulated genes in the samples treated with the peptides. The list of the 100 most affected genes generated by microarray analysis included 35 genes which are in the heat map indicated as lower expressed genes, 23 of those can be identified to the family of immunoglobulins whose members presumably act as adhesion molecules [3]. The lower expression of these "adhesion molecules" may, in view of the fact that both examined differently treated cell cultures were confluent, indicate a fully closed adhesion phase and advanced differentiation of the cells supplied with the peptides. Of particular importance is the significantly higher expression of BMP 2, osteopontin (SPP1), MMP 1, MMP 9 and stanniocalcin 1.

BMP 2 (bone morphogenetic protein 2) is a member of the "transforming growth factor-beta" superfamily, functions as a homodimer and especially induces bone formation [15, 37]. For several decades, BMP 2 stands in the focus of many studies as an additive for bone substitute materials because its osteoinductive effect is verified. However, in many studies BMP 2 is added separately to the materials, which in relation to the concentration and its effect on bone cells can certainly cause problems. By the addition of the EP the cell's own production of BMP 2 increases and therefore represent a very interesting and important approach for bone replacement materials research.

M. Mizuno and Y. Kuboki showed that osteopontin (OPN or SPP1) is synthesized during the differentiation of osteoblasts and subsequently incorporated into the mineralized collagen matrix [25]. During the formation of new bone OPN regulates the crystal growth of apatite. Osteopontin is also involved in the adhesion of osteoclasts on the mineralized bone matrix and is known to be a specific immunocytochemical bone marker [17, 25, 45]. The demonstrated higher gene expression analysis and the increased expression of OPN is emphasized at this point.

Another significantly higher expressed gene in the cultures treated with the peptides is stanniocalcin 1 (STC 1). In osteoblasts, osteoclasts, myoblasts and endothelial cells STC 1 can affect the calcium and phosphorus regulation and so on bone mass formation, structure, as well as angiogenesis. During bone development, it is found in chondrocytes and osteoblasts [13]. Furthermore, it stimulates according to a study by Yoshiko et al. the osteogenic differentiation of rat osteoblasts too [42].

In addition, the two tissue remodelling matrix metalloproteinases MMP 1 and MMP 9 could be detected with an up-regulated expression in the peptide treated group by qRT-PCR. These are essential factors during the physiological collagen degradation and the matrix remodelling during embryonic development. MMP 1 transcribes an enzyme that specifically causes the degradation of interstitial collagens I, II and III. It is synthesized in the bone by stromal fibroblasts, osteoblasts and osteoclasts, and together with MMP 8 it is the only enzyme that could cleave collagen type I in the not-denatured state and at neutral pH-values. This mechanism plays an important role in remodelling of existing bone [19]. MMP 9 encodes an enzyme that is responsible for the degradation of different collagens and is therefore an important factor in the physiological bone remodelling process [6].

## Conclusions

In this study, the efficacy of differently concentrated eggshell extracted peptides (EP) and their combination with hyaluronic acid was analyzed on bovine osteoprogenitors and human osteoblasts in vitro. The increase in proliferation, as well as differentiation of bovine osteoprogenitor cells and their cell-controlled mineralization, was significant for all peptide concentrations, but highest for EP II (0.5 g/L) and HAEP. The outstanding results of Richardson staining, as well as histochemical staining of bone-specific matrix proteins of collagen type I, osteonectin, osteopontin and osteocalcin after treatment with EP in a concentration of 0.5 g/L, underline the beneficial effects of eggshell peptides on bone regeneration. The mineral content after 4 weeks, as a key indicator for successful osteoblast differentiation, could be increased with incorporation of 2.5 g/L EP into hyaluronic acid to a factor of 286 compared to the untreated culture. The main important genes in bone formation and regeneration such as BMP 2, osteopontin, STC1 and the matrix metalloproteinases 1 and 9 were upregulated, detected by microarray and qRT-PCR during culture of primary human osteoblasts treated with 0.5 g/L EP. The influence of different matrix proteins on the implementation and incorporation of calcium during eggshell formation as well as the initiation of embryonic bone formation in chicks is known. Based on our findings, putamen ovi peptides also have a beneficial effect on bone formation and regeneration and seem to be predestinated for the usage as stimulators or mediators in the formation of growth factors for bone remodelling. A major factor for the applications is furthermore the immune response, which can only be investigated in vivo. The contraindicative influence of hyaluronan on eggshell peptides (HAEP vs. EP II) already shows the complex interaction, why future work should focus on in vivo trials. Considering the limitations of the study, the hyaluronic acid-embedded peptides of putamen ovi reveal their full potential in supporting bone formation with a high mineralisation induction as well as a significant osteogenic cell differentiation.

## Data Availability

The datasets used and/or analysed during the current study are available from the corresponding author on reasonable request.

## Abbreviations

EP: Eggshell peptides
qRT-PCR: Quantitative real-time polymerase chain reaction
BMP 2: Bone morphogenetic protein 2
OPN/SPP1: Osteopontin
HA: Hyaluronan / hyaluronic acid
HAEP: Eggshell peptides supplemented with hyaluronic acid
PBS: Phosphate buffered saline
HCl: Hydrochloric acid
RNA: Ribonucleic acid
cDNA: Complementary deoxyribonucleic acid
ANOVA: Analysis of variance
MMP: Matrix metallopeptidase
STC: Stanniocalcin
HPRT: Hypoxanthine phosphoribosyltransferase
GAPDH: Glyceraldehyde-3-phosphate dehydrogenase
